# Cranial findings detected by second‐trimester ultrasound in fetuses with myelomeningocele: a systematic review

**DOI:** 10.1111/1471-0528.16496

**Published:** 2021-01-03

**Authors:** Y Kunpalin, J Richter, N Mufti, J Bosteels, S Ourselin, P De Coppi, D Thompson, AL David, J Deprest

**Affiliations:** ^1^ Elizabeth Garrett Anderson Institute for Women's Health University College London London UK; ^2^ Department of Development and Regeneration, Biomedical Sciences KU Leuven Leuven Belgium; ^3^ Department of Obstetrics and Gynaecology University Hospitals Leuven Leuven Belgium; ^4^ Cochrane Belgium Belgian Centre for Evidence‐Based Medicine (Cebam) Leuven Belgium; ^5^ School of Biomedical Engineering & imaging Sciences King's College London London UK; ^6^ Department of General Paediatric Surgery Great Ormond Street Institute of Child Health University College London London UK; ^7^ Department of Paediatric Neurosurgery Great Ormond Street Institute of Child Health University College London London UK

**Keywords:** Chiari II malformation, fetal surgery, myelomeningocele, prenatal, spina bifida, spina bifida aperta, spinal dysraphism, ultrasonography

## Abstract

**Background:**

Abnormal intracranial findings are often detected at mid‐trimester ultrasound (US) in fetuses with myelomeningocele (MMC). It is unclear whether these findings constitute a spectrum of the disease or are an independent finding, which should contraindicate fetal surgery.

**Objective:**

To ascertain the spectrum and frequency of US‐detected cranial findings in fetuses with MMC.

**Search strategy:**

MEDLINE, Embase, Web of Science and CENTRAL were searched from January 2000 to June 2020.

**Selection criteria:**

Study reporting incidence of cranial US findings in consecutive cases of second‐trimester fetuses with MMC.

**Data collection and analysis:**

Publication quality was assessed by Newcastle–Ottawa Scale (NOS) and modified NOS. Meta‐analysis could not be performed as a result of high clinical diversity and study heterogeneity.

**Main results:**

Fourteen cranial US findings were reported in 15 studies. Findings in classic Chiari II malformation (CIIM) spectrum included posterior fossa funnelling (96%), small transcerebellar diameter (82–96%), ‘banana’ sign (50–100%), beaked tectum (65%) and ‘lemon’ sign (53–100%). Additional cranial findings were small biparietal diameter (BPD) and head circumference (HC) (<5th centile; 53 and 71%, respectively), ventriculomegaly (45–89%), abnormal pointed shape of the occipital horn (77–78%), thinning of the posterior cerebrum, perinodular heterotopia (11%), abnormal gyration (3%), corpus callosum disorders (60%) and midline interhemispheric cyst (42%).

**Conclusions:**

We identified 14 cranial findings by second‐trimester US in fetuses with MMC. The relatively high incidence of these findings and their unclear prognostic significance might not contraindicate fetal surgery in the case of normal fetal genetic testing. Some cranial findings may independently affect postnatal outcome, however. Long‐term detailed follow‐up is required to investigate this.

**Tweetable abstract:**

A high rate of cranial abnormalities found on second‐trimester ultrasound in fetuses with myelomeningocele.

## Tweetable abstract

A high rate of cranial abnormalities found on second‐trimester ultrasound in fetuses with myelomeningocele.

## Introduction

Myelomeningocele (MMC) is a severe congenital malformation of the central nervous system that results from incomplete closure of the neural tube during the third to fourth week of embryonic development.^1^ The disease is characterised by a malformation of the spinal cord associated with a defect in the posterior spinal elements and overlying skin. MMC is commonly detected on ultrasound examination in the first or second trimester.^1^ Without tissue coverage, the neural placode is exposed to the uterine environment, rendering it vulnerable to secondary damage throughout gestation leading to lifelong sensory and motor impairments, bowel and bladder dysfunction, and orthopaedic disabilities.^2^ In addition to the spinal lesion, characteristic changes in brain development occur that are presumed to result from abnormal molecular genetic mechanisms and mechanical consequences of cerebrospinal fluid (CSF) leakage.^3^ These brain changes constitute the classic Chiari II malformation (CIIM). Well‐recognised features of CIIM include a small posterior fossa with an upward and downward herniation of the cerebellar and vermian structures, through the tentorial incisura and the foramen magnum, deformation of the brainstem structures, including pons, medulla and fourth ventricle, as well as abnormal cerebellar morphology.^3^, ^4^, ^5^ Additional cranial features associated with MMC comprise colpocephaly, commissural anomalies, neuronal migration and cortical organisation disorders.^5^, ^6^, ^7^ Not all of these findings may be detectable by ultrasound or evident before birth.

Both the spinal and cranial features of MMC are known to progress during prenatal life, and this has been the rationale behind in utero closure of the defect, which is now proven to improve several postnatal outcomes when compared with standard postnatal surgery.^8^, ^9^, ^10^, ^11^, ^12^ One of the exclusion criteria for fetal surgery is the presence of fetal anomalies unrelated to MMC.^8^ Therefore, a comprehensive assessment of the fetus, with particular emphasis on the brain, is mandatory before surgery. This imaging policy has resulted in the identification of a range of brain abnormalities on prenatal ultrasound, varying from subtle to severe. Some of these findings are similar to those previously described in postnatal series.^5^, ^13^

The identification of these abnormalities, which often outlie the classic features of CIIM, leads to a dilemma as to whether these are part of the disease spectrum or whether these are additional independent features with their own prognosis, potentially representing a contraindication to fetal surgery. To our knowledge, this remains unclear. The aim of this review was to determine systematically the spectrum and frequency of cranial ultrasound findings associated with MMC that can be detected in the second trimester (at 14–28 weeks of gestation).

## Methods

This systematic review is reported according to the Preferred Reporting Items for Systematic Review and Meta‐analyses (PRISMA) guidelines (www.prisma‐statement.org).^14^ We have published our protocol prospectively in the Prospero registry (CRD42019139703). This research is funded by an Innovative Engineering for Health award from the Wellcome Trust (WT101957) and an Engineering and Physical Sciences Research Council (EPSRC) grant (NS/A000027/1). There was no patient and public involvement in this review.

### Search strategy

We conducted an electronic literature search in MEDLINE (PubMed), Embase, Web of Science and The Cochrane Central Register of Controlled Trials (CENTRAL) from 2000 until June 2020. This is to avoid the inclusion of older less technically advanced studies. The search strategy combined Medical Subject Headings (MeSH) terms and keywords, as shown in Appendix S1. We used endnote x9 (Clarivate Analytics, Philadelphia, PA, USA) to remove duplicate studies based on names of the authors, titles and year of publication. We performed ‘snowballing’ by hand searching the reference lists of topic‐related reviews and eligible papers to retrieve additional relevant articles.

### Study selection

Titles and abstracts were independently screened and selected for relevance by two reviewers (YK and JR). Selected studies were checked for eligibility by the two reviewers based on the eligibility criteria described below. Any disagreement was resolved through discussion, and if this was not possible we sought arbitration from a third reviewer (NM). In the case of overlapping studies, only the most recent publication was included.

### Eligibility criteria

Studies were sought that reported the prevalence of intracranial findings in fetuses with MMC detected on ultrasound examination during the second trimester (at 14–28 weeks of gestation). Studies were included only if they clearly stated that the authors retrieved all consecutive cases of MMC from their database. No restriction was made based on fetal chromosomal abnormalities or additional extracranial anomalies. Retrospective/prospective cohort studies, observational studies, cross‐sectional studies and randomised controlled trials were considered. Studies published in non‐English language or as case reports or reviews were excluded. Those without full‐text accessibility were also excluded.

### Data extraction and analysis

A predefined form was created by the reviewers before data extraction. Extracted information included participant characteristics, technical aspects on image acquisition and study methodology (Appendix S2). Participant characteristics included maternal and gestational age at the time of evaluation (range, mean, median), associated anomalies or chromosomal abnormalities, total number of examinations and prevalence of all intracranial findings. Technical aspects included details regarding the ultrasound hardware used, the ultrasound probe and level of experience and/or numbers of sonographers. Methodological information included study design and primary outcomes.

The incidence of intracranial findings from each study were reported as a percentage (number of patients with a specific intracranial finding/total number of patients in each study × 100). Cranial findings were categorised into CIIM spectrum and additional cranial findings outside CIIM spectrum, according to previous publications.^15^, ^16^, ^17^, ^18^, ^19^, ^20^, ^21^ In this publication, ‘ultrasound sign’ refers to parameters that were detected by ultrasound only, whereas ‘cranial abnormality’ refers to parameters that can be validated across all imaging and pathological modalities.

### Quality appraisal

Both YK and JR assessed the methodological quality independently using the Newcastle–Ottawa Scale (NOS) for cohort studies and the modified NOS for cross‐sectional studies. Three domains (selection, comparability and outcome) were rated for risk of bias, categorised as unsatisfactory, satisfactory, good and very good quality in cross‐sectional studies, and poor, fair and good quality in cohort studies.^22^, ^23^ Discrepancies between the reviewers were resolved through discussion, and when this was not possible by consensus following arbitration from the third reviewer (NM).

## Results

### Search result

**Figure 1:**
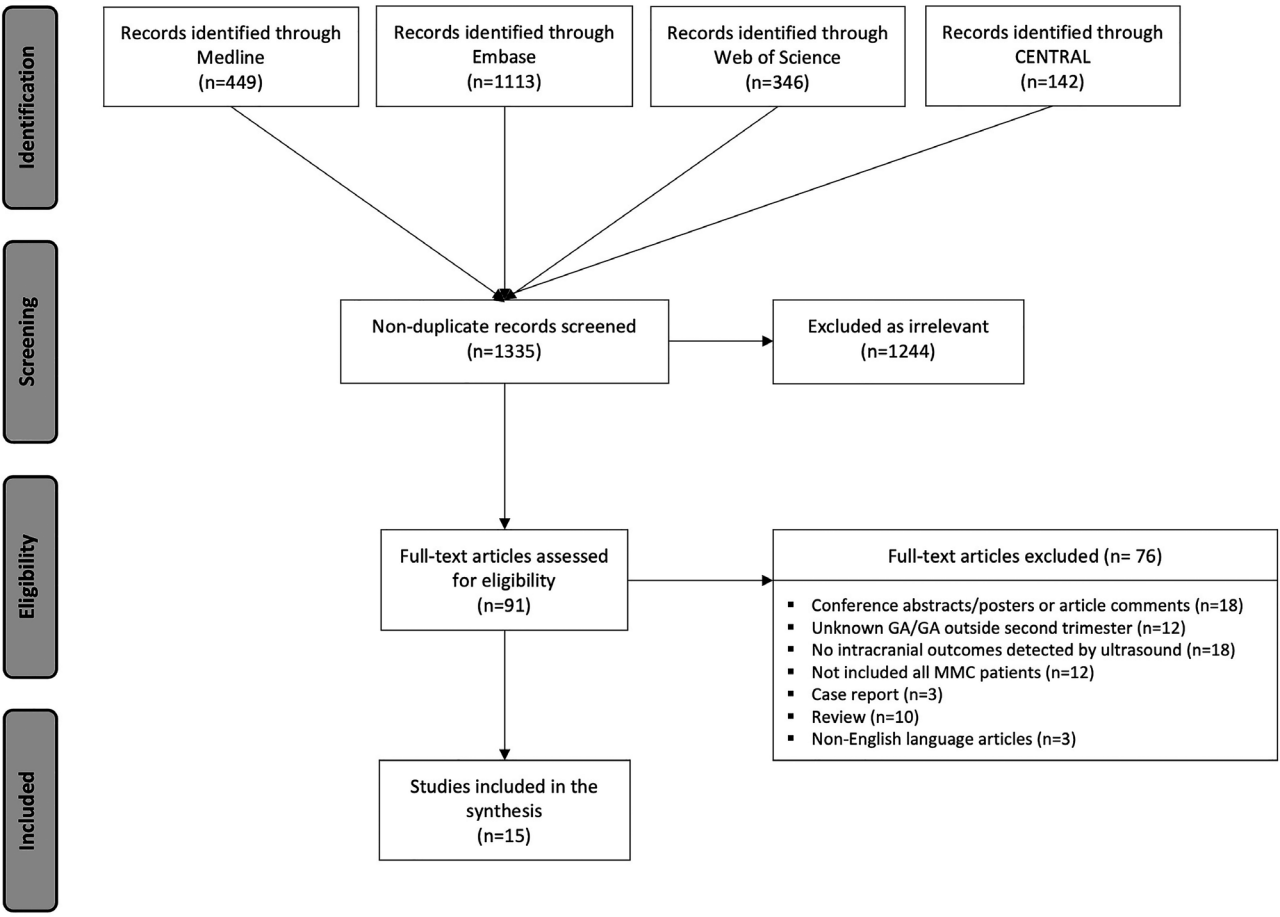
Flow diagram of illustrated study selection (adapted from Preferred Reporting Items for Systematic Reviews and Meta‐Analysis, PRISMA).^15^

The electronic literature search yielded 2050 studies published between January 2000 and June 2020; 715 duplicate studies were removed. Titles and abstracts of the remaining 1335 studies were screened and 1244 studies were excluded as irrelevant. A total of 91 studies were reviewed as full text, from which 15 studies were eventually included. Reasons for exclusion were: insufficient information (conference abstract and poster presentations or article comments; 18/76, 24%), no reporting of intracranial findings detected by ultrasound (18/76, 24%), inadequate study design (case reports or reviews; 13/76, 16%), no reporting of gestational age (GA) or GA outside second trimester (<14 or >28 weeks of gestation; 12/76, 16%), not including all cases of MMC (12/76; 16%), and non‐English articles (3/76, 4%) (Figure 1).

### Characteristics and quality of included studies

The characteristics of the included studies, such as study design, primary outcomes, population studied, gestational age at ultrasound examination, ultrasound machine/probe and sonographer, are shown in Table S1. Four studies excluded fetuses with chromosomal abnormalities or excluded fetuses with additional structural anomalies (4/15, 27%); three studies excluded both conditions (3/15, 20%). As a result of the high clinical diversity and significant statistical heterogeneity, we refrained from performing a meta‐analysis. Diversities include primary outcomes, inclusion/exclusion of cases with chromosomal abnormalities or additional structural anomalies and the competency level of sonographers.

The quality assessment is presented in Tables S2 and S3. Overall, the quality of cross‐sectional studies was better than that of cohort studies, with most having high quality (12/15, 80%) (NOS for cohort study, good; modified NOS for cross‐sectional study, satisfactory to good). For studies with a high risk of bias, this was linked to a lack of confounding control methods.

### Cranial findings in fetuses with MMC

Table 1 summarises the range of intracranial findings that were identified in the included studies: small biparietal diameter (BPD), small head circumference (HC), abnormal brain parenchyma and corpus callosum, ventriculomegaly, abnormal shape and position of the lateral ventricles, abnormal shape of the midbrain, ‘lemon’ sign, funnelling of the posterior fossa and abnormal cerebellum.

**Table 1:** Cranial findings in fetuses with myelomeningocele

Intracranial findings	Name of first author	GA at examination (weeks)	*n*/*N* (%)
Funnelling of posterior fossa^a^	D'Addario, 2008	18–28	47/49 (96)
Small transcerebellar diameter (TCD)^b^	Cuppen, 2015	16–26	42/51 (82)
	D'Addario, 2008	18–28	47/49 (96)
Banana sign^c^	Appasamy, 2006	16–23	6/12 (50)
	Bahlmann, 2015	18–23	608/627 (97)
	D'Addario, 2008	18–28	46/49 (94)
	Fleurke‐Rozema, 2014	18–24	16/16 (100)
	Ghi, 2006	20.3 (mean)	53/53 (100)
	Munoz, 2019	19.2 (mean)	40/45 (89)
	Ramin, 2002	19–22	24/33 (73)
Abnormal shape of midbrain^d^	Callen, 2009	17–24	46/71 (65)
Lemon sign^e^	Appasamy, 2006	16–23	7/12 (53)
	Bahlmann, 2015	18–23	549/627 (88)
	D'Addario, 2008	18–28	29/46 (53)
	Fleurke‐Rozema, 2014	18–24	57/71 (80)
	Ghi, 2006	20.3 (mean)	53/53 (100)
	Munoz, 2019	19.2 (mean)	41/45 (91)
	Ramin, 2002	19–22	30/33 (91)
Small biparietal diameter (BPD)^f^	Bahlmann, 2015 (<5th centile)	18–23	335/627 (53)
Small head circumference (HC)^g^	Bahlmann, 2015 (<5th centile)	18–23	442/627 (71)
Perinodular heterotopia^h^	Maurice, 2020	20–26	8/70 (11)
Gyration disorders^i^	Maurice, 2020	20–26	2/70 (3)
Abnormal corpus callosum^j^	Maurice, 2020	20–26	42/70 (60)
Ventriculomegaly^k^	Appasamy, 2006	16–23	7/12 (58)
	Bahlmann, 2015	18–23	282/627 (45)
	Cuppen, 2015	16–26	39/74 (53)
	D'Addario, 2008	18–28	40/49 (82)
	Fleurke‐Rozema, 2014	18–24	41/71 (58)
	Ghi, 2006	20.3 (mean)	34/53 (64)
	Maurice, 2020	20–26	39/70 (56)
	Munoz, 2019	19.2 (mean)	40/45 (89)
	Oliver, 2019	18–25	232/350) (66)
	Ramin, 2002	19–22	25/33 (76)
Abnormal shape of lateral ventricle^l^	Callen, 2008	17–24	49/63 (78)
	Wax, 2009	19.2 (mean)	17/22 (77)
Abnormal position of lateral ventricle^m^	Filly, 2010	18–26	25/25 (100)
Interhemispheric arachnoid cyst^n^	Wong, 2009	17–24	30/71 (42)

^a^Clivus–supraocciput angle <72°; ^b^TCD <10th percentile according to gestational age; ^c^anterior curvature of cerebellum; ^d^beaked, elongated tectum of midbrain; ^e^scalloping of frontal bones; ^f^BPD <3rd or <5th percentile according to gestational age; ^g^HC <3rd or <5th percentile according to gestational age; ^h^perinodular or subependymal heterotopia; ^i^gyration disorders; ^j^abnormal corpus callosum, anomalies of the corpus callosum, i.e. absent, incomplete, short or stretched corpus callosum; ^k^maximum atrial width of ≥10 mm; ^l^pointed occipital horn of lateral ventricle; ^m^short distance from posterior edge of occipital horn to inner edge of occipital bone; ^n^interhemispheric cyst, pineal cysts and cavum veli interpositi.

### Classic CIIM spectrum

**Figure 2:**
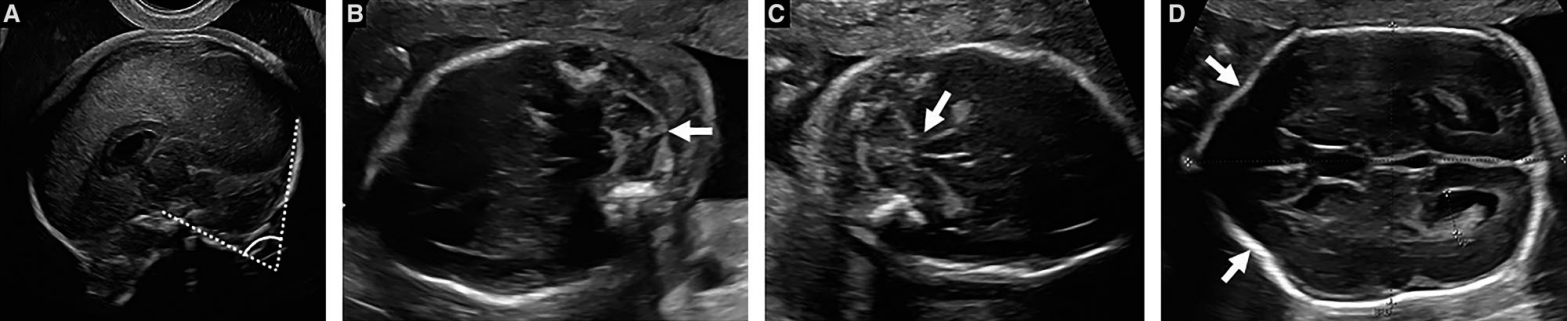
Classic Chiari II malformation findings detected by ultrasound in fetuses with open spina bifida: (A) midsagittal view demonstrating a clivus–occipital bone angle of 69°; (B) transcerebellar view showing a ‘banana’ sign of the cerebellum and an effaced cisterna magna; (C) transcerebellar view showing prominent beaking and elongation of the tectum; (D) transventricular view demonstrating the scalloping of the frontal bones of fetal skull, known as the ‘lemon’ sign. Reprinted with permission of the UZ Leuven, Leuven, Belgium.

There were nine studies reporting cranial findings considered to be in the CIIM spectrum. These included abnormal shape of the posterior fossa, abnormalities of the cerebellum, elongated quadrigeminal plate of the midbrain and a scalloping of the frontal bones of the fetal skull. D'Addario studied the shape of the posterior fossa and found that 96% of the fetuses examined had a small clivus–occipital bone angle (<72°).^24^ The angle is measured on a mid‐sagittal plane of the fetal skull between a line drawn on the occipital bone and another drawn on the clivus bone^25^ (Figure 2).

Cerebellar abnormalities include abnormal size, small transcerebellar diameter (TCD, <10th centile), abnormal shape and anteriorly effaced cerebellum, known as the ‘banana’ sign (Figure 2).^21^ One study reported both the size and the shape of the cerebellum, another study reported only a small TCD and the remaining six studies only reported the banana sign. The small TCD was present in 82–96% of the fetuses examined.^24^, ^26^ The incidence of the banana sign varied between series, ranging from 50 to 100%.^24^, ^27^, ^28^, ^29^, ^30^, ^31^, ^32^ Bahlmann et al. found no association between ventricle size and the incidence of a banana‐shape cerebellum.^28^

One study reported an abnormal shape of the quadrigeminal plate or tectum in 65% of cases (Figure 2). It appeared as a beaked, elongated tectum instead of its normal, square–round shape at <24 weeks of gestation. Both GA and ventricle size did not have any impact on tectal shape; however, the severity of cerebellar abnormalities was correlated with the presence of a beaked tectum.^33^

**Figure 3:**
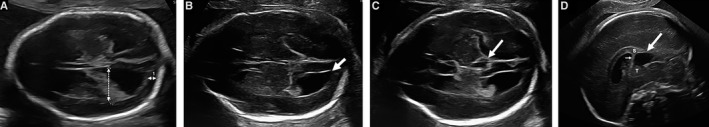
Additional cranial findings detected by ultrasound in fetuses with open spina bifida: (A) transventricular view showing a mild ventriculomegaly with a short distance between the posterior edge of the occipital horn and the occipital bone (calliper 1); (B) transventricular view showing a normal size lateral ventricle with a pointed occipital horn; (C) transventricular view demonstrating a midline anechoic interhemispheric cyst; (D) mid‐sagittal view of the same fetal brain showing a midline cyst situated below the splenium of the corpus callosum (S) and the column of the fornix (small arrow), and posterior to the thalamus (T). Reprinted with permission of the UZ Leuven, Leuven, Belgium.

The most prevalent finding in fetuses with MMC was the so‐called lemon sign (Figure 3), defined as a scalloping of the frontal bones of the fetal skull.^21^ The incidence ranged from 53 to 100% between 14 and 28 weeks of gestation.^24^, ^27^, ^28^, ^29^, ^30^, ^31^, ^32^ The incidence of the lemon sign was associated with the presence of ventriculomegaly (89 versus 74%, *P* = 0.01), but not with the level of the MMC defect.^28^

### Additional cranial ultrasound findings

The head size of fetuses with MMC was found to be small, both in terms of BPD and HC. Using the fifth centile as a cut‐off point, the incidences of small BPD and HC were 53 and 71%, respectively.^28^ As gestation advanced, or when there was ventriculomegaly, a smaller head size was more common.^28^

Recently, Maurice et al. reported that 60 and 14% of fetuses with MMC had abnormal corpus callosum and brain parenchyma findings on ultrasound assessment, respectively. Abnormal corpus callosum included stretched corpus callosum in 7%, short but complete corpus callosum in 35%, short and incomplete corpus callosum in 17% and a total absence of the structure in 1%. In the same publication, the authors also reported abnormalities of cortical development. This comprised perinodular heterotopia (PNH) and abnormal gyral patterning, which were seen in 11 and 3% of MMC cases, respectively.^34^

A total of ten studies reported the incidence of ventriculomegaly in the second trimester. The incidence of ventriculomegaly, defined as lateral ventricular size of ≥10 mm, ranged from 45 to 89% of cases (Figure 3).^24^, ^35^ Of those, mild‐to‐moderate and severe ventriculomegaly were documented in 85 and 15%, respectively.^26^

Regarding the shape of the lateral ventricles, an abnormal ultrasound finding was identified in the occipital horns: the normal round shape of the structure was substituted by an abnormal angulated/pointed contour^36^, ^37^ (Figure 3). When the posterior lateral ventricles were adequately visualised, the finding was found in 77–78% of fetuses between 17 and 24 weeks of gestation.^36^, ^37^ Gestational age, cerebellar abnormalities or level of MMC defect had no association with the pointed shape of the ventricles.^36^, ^37^ Filly and colleagues described a diminished thickness of the cerebral occipital lobe between the occipital horn of the lateral ventricles and the occipital bone in all fetuses with MMC, resulting in the impression of ‘too‐far‐back’ lateral ventricles.^38^

Wong et al. described abnormal midline cystic structures in fetuses with MMC. Between 17 and 24 weeks of gestation, 42% of fetuses with MMC had interhemispheric cysts. These cysts included arachnoid cysts, pineal cysts and cyst‐like structures of the cavum veli interpositi (CVI) (Figure 3). Gestational age, the degree of cerebral abnormalities or ventriculomegaly were not related to the incidence of the cysts.^39^

## Discussion

### Main findings

Overall, 15 studies (published between 2000 and June 2020) were identified that reported 14 cranial abnormalities detected by ultrasound in the second trimester. As a result of the high clinical and methodological heterogeneity, a meta‐analysis was not deemed appropriate.

Of the 14 cranial ultrasound findings related to MMC, around a third fall in the CIIM spectrum. Cranial abnormalities classified in the CIIM spectrum included funnelling of posterior fossa, small transcerebellar diameter, banana‐shaped cerebellum, beaked tectum and lemon sign. Additional cranial ultrasound findings comprised small head size, abnormal corpus callosum and brain parenchyma, ventriculomegaly, pointed occipital horns of lateral ventricle, thin occipital lobe and interhemispheric cyst.

### Strengths and limitations

Limitations of this systematic review are the variety of primary outcomes in the studies included, the nature of observational studies, and the low proportion of studies that provide information about the exclusion of fetuses with chromosomal abnormalities and/or additional structural anomalies. Lastly, we only examined publications written in English.

To our knowledge, this is the first systematic review of studies reporting cranial abnormalities that can be detected by ultrasound during the second trimester in fetuses with MMC. We have identified a range of abnormal brain findings in fetuses with MMC, other than the well‐known banana and lemon signs. Some of the findings are outside the spectrum of CIIM and cannot be simply explained by mechanical pathogenesis.

### Interpretation

During the development of the central nervous system (CNS), the normal expression of different genes specific to different CNS regions is vital to ensure neural tube closure and cranial skull development.^3^ In the case of MMC, abnormal genetic expression and reduction in ventricular pressure from CSF leakage results in a small cranial vault, particularly in the posterior fossa.^3^, ^4^ D'Addario finds that almost all of the fetuses with MMC in their study had a small clivus–supraoccipital angle (<72°), indicative of a reduced posterior fossa size.^24^, ^25^ Growth of the supratentorial compartment is also compromised in fetuses with MMC, as reflected by an inward concavity of the frontal bones, known as the lemon sign.^4^

It is suggested that the small size of the posterior fossa is inadequate to accommodate the growing hindbrain, which as a result herniates cranially and caudally, causing CIIM.^4^ The majority of the anatomical features of CIIM are thought to be a consequence of this growth‐related deformation: for example, cranial herniation results in an elongation of the tectal plate, which was seen in almost two‐thirds of fetuses with MMC,^33^ whereas caudal displacement of the cerebellar vermis and tonsil can be detected as a banana‐shaped cerebellum by ultrasound.

Hindbrain herniation associated with CIIM may compromise CSF outflow from the fourth ventricle, which is often small and compressed, resulting in ventricular dilatation. Altered CSF flow through the ventricular system might also be responsible for the appearance of CVI.^4^ CVI is the dilatation of the posterior third ventricle, presenting as a regular border, anechoic, interhemispheric cyst below the splenium of the corpus callosum.^39^, ^40^ This abnormality is common in fetuses with MMC because the anterior part of the third ventricle cannot dilate owing to an adhesion known as massa intermedia, which are also prevalent in MMC cases.^5^, ^41^

The majority of fetuses affected by MMC have a small head size, in part because of the effect of chronic CSF loss through the spinal defect and the resultant lack of intracranial tension required to maintain head growth. Fetal surgery stops the CSF egress and has been shown to permit the normalisation of the head contour.^42^

In terms of MMC diagnostic value, both lemon and banana signs are invaluable signs for the diagnosis of MMC at <24 weeks of gestation (lemon sign, sensitivity 98%, specificity 99%; banana sign, sensitivity 96%, specificity 100%).^16^ Unlike the banana sign, the lemon sign resolves after 24 weeks of gestation as the skull becomes ossified and strengthens.^43^ Other cranial findings that might aid in MMC diagnosis during the second trimester are a pointed occipital horn of the lateral ventricle and a clivus–supraoccipital angle of <72°. A pointed occipital horn is relatively prevalent in fetuses with MMC but it is not a pathognomonic sign.^37^ As the lateral ventricle should be examined in every fetus, the observation of the occipital horn is feasible, and this sign may be of benefit if cases of MMC present later in gestation, at a time when the lemon sign disappears.^28^, ^29^ A small clivus–supraocccipital angle was found to persist through the second trimester in almost all of the cases.^24^, ^25^ A mid‐sagittal plane of the fetal brain is needed to measure the angle, however, and this may make the sign impractical as this plane is not routinely incorporated into the anomaly scan.^44^

In the second trimester, ventriculomegaly is one of the most important factors to predict the postnatal outcome of fetuses with MMC, particularly the need for a ventriculoperitoneal (VP) shunt. Both the size and the early onset of ventriculomegaly are positively associated with shunt requirement after birth.^45^, ^46^ Other postnatal cranial abnormalities, such as beaking of the midbrain tectum, callosal anomalies and PNH, are suggested to have functional implications for individuals with MMC. In a postnatal MRI study, the degree of tectal beaking was shown to be correlated with the severity of nystagmus.^47^ Beaked tectum was present in more than half of the children with MMC.^47^, ^48^ Postnatal MRI studies also reveal an association between the volume and integrity of the corpus callosum and intelligence quotient (IQ) level, delayed reaction time to stimuli, slower bimanual coordination and poor idiom comprehension in children with MMC.^49^, ^50^, ^51^

Perinodular heterotopia is strongly associated with other cortical or cerebral malformations that often cannot be detected by second‐trimester ultrasound.^52^, ^53^ After birth, common clinical manifestations of PNH are epilepsy and cognitive disorders, present in 80% and 20–60% of cases, respectively. The incidence, severity and age at onset of these clinical signs depends upon the presence of cranial or genetic anomalies in addition to PNH.^53^, ^54^ Although there are currently no data to determine whether the co‐existence of PNH and MMC carries a worse prognosis, this seems likely when considering that PNH itself has an unfavourable prognosis.

## Conclusion

This systematic review has identified 14 sonographic cranial findings detected during the second trimester in fetuses with MMC, of which one‐third are in the CIIM spectrum. The majority of these findings are in keeping with what is known from postnatal imaging studies and can be attributed to the mechanical consequences of an MMC lesion. Some abnormalities may not be so attributable to MMC, however, and yet their frequency would indicate that they are likely to be part of the MMC disease spectrum. With the increasing screening of MMC pregnancies for suitability for fetal surgery, it is important to better understand the diversity of intracranial findings that are present in this population. There is a need for a standardised approach to characterise brain and spinal abnormalities in fetuses with MMC. In addition, detailed neurocognitive outcomes for children with MMC are required to ascertain the potential clinical significance of these ‘additional’ ultrasound findings.

### Disclosure of interests

None declared. Completed disclosure of interests form is available to view online as supporting information.

### Contribution to authorship

YK, JB, SO, PDC, ALD and JD designed the study. YK, JR and NM performed the review and extracted the results. YK, JR and DT drafted the article. All authors contributed to editorial changes.

### Funding

This research was funded by an Innovative Engineering for Health award by the Wellcome Trust (WT101957) and an Engineering and Physical Sciences Research Council (EPSRC) grant (NS/A000027/1). JD is supported by the Great Ormond Street Hospital for Children's Charity. JB is supported by the Department of Development and Regeneration, Cluster Woman and Child. ALD is supported by the National Institute for Health Research University College London Hospitals Biomedical Research Centre. PC is supported by the National Institute for Health Research Great Ormond Street Hospital Biomedical Research Centre.

## Supporting information

**Table S1.** Characteristics of included studies.
**Table S2.** Quality appraisal for cohort study assessed by Newcastle–Ottawa scale.
**Table S3.** Quality appraisal for cross‐sectional study assessed by modified Newcastle–Ottawa scale.

**Appendix S1.** Search strategy.
**Appendix S2.** Characteristics of included studies and results.

Supplementary Material

Supplementary Material

Supplementary Material

Supplementary Material

Supplementary Material

Supplementary Material

Supplementary Material

Supplementary Material

Supplementary Material
