# Supplementary material for: Cranial findings detected by second‐trimester ultrasound in fetuses with myelomeningocele: a systematic review
Source: BJOG. 2021 Jan 3;128(2):366–74. doi: 10.1111/1471-0528.16496 (PMC8436766; doi:10.1111/1471-0528.16496)
Supplement: Supplementary file 1 — Table S1. Characteristics of included studies. Table S2. Quality appraisal for cohort study assessed by Newcastle–Ottawa scale. Table S3. Quality appraisal for cross‐sectional study assessed by modified Newcastle–Ottawa scale. [file BJO-128-366-s010.pdf]

**Table S1.** Characteristics of included studies

| Study | Study design | Primary outcome | Population | Gestational age at examination | Chromosomal abnormalities exclusion | Additional anomalies exclusion | Ultrasound machine | Ultrasound probe | Level of sonographer |
|---|---|---|---|---|---|---|---|---|---|
| **Appasamy, 2006 (21)** | Retrospective cohort | Compare prenatal and postnatal US/MRI findings on head signs and lesion levels in fetuses with MMC | Prenatal & postnatal diagnosis | 16-23 weeks | NA | NA | Varies | NA | NA |
| **Bahlmann, 2015 (22)** | Cross-sectional (retrospective analysis) | Determine the incidence of secondary cranial signs in fetuses with MMC | Prenatal diagnosis | 18-23 weeks | Yes | NA | Machines used below† | NA | US specialists |
| **Callen, 2008 (30)** | Cross-sectional (retrospective analysis) | Determine the incidence of pointed deformity of occipital horns of lateral ventricles in fetuses with MMC | Prenatal diagnosis | 17-34 weeks | NA | NA | NA | 2-4-MHz vector /2-6-MHz curved array transducer | Single US specialist |
| **Callen, 2009 (27)** | Cross-sectional (retrospective analysis) | Determine the incidence of prominent beaking and elongated tectum in fetuses with MMC | Prenatal diagnosis | 17-34 weeks | NA | NA | NA | 2-4-MHz vector /2-6-MHz curved array transducer | Single US specialist |
| **Cuppen, 2015 (20)** | Cross-sectional (retrospective analysis) | Assess fetal BPD and HC during second trimester in fetuses with MMC | Prenatal diagnosis | 16-26 weeks | NA | NA | NA | NA | US specialists |
| **D'Addario, 2008 (17)** | Cross-sectional (retrospective analysis) | Compare diagnostic accuracy of lemon sign, ventriculomegaly, effaced cisterna magna, small cerebellum, small clivo-supraoccipital angle and spinal defect in detecting fetuses with MMC | Prenatal diagnosis | 18-28 weeks | NA | NA | NA | NA | NA |
| **Filly, 2010 (32)** | Cross-sectional (retrospective analysis) | Describe the abnormal more posterior position of occipital horn of the lateral ventricle in fetuses with MMC | Prenatal diagnosis | 18-30 weeks | NA | NA | NA | 2-4-MHz vector /2-6-MHz curved array transducer | NA |
| **Fleurke-Rozema, 2014 (23)** | Retrospective cohort | Assess the impact of mid-trimester ultrasound on pregnancy outcomes of MMC | Prenatal & postnatal diagnosis | 18-24 weeks | NA | NA | NA | NA | NA |
| **Ghi, 2006 (24)** | Cross-sectional (retrospective analysis) | Identify criteria important to differentiate closed from MMC | Prenatal & postnatal diagnosis | 16-34 weeks | NA | Yes | Varies | NA | NA |
| **Maurice, 2020 (28)** | Retrospective cohort | To examine cerebral anomalies related to MMC besides Chiari II malformation | Prenatal & postnatal diagnosis | 20-26 weeks | Yes | Yes | Canon Aplio 400 unit | Vector and curved array or transvaginal transducer | Single US specialist |
| **Munoz, 2019 (25)** | Retrospective cohort | Identify maternal and fetal characteristics associated with pregnancy outcomes of MMC | Prenatal diagnosis | 20.3 weeks* | Yes | Yes | NA | NA | NA |
| **Oliver, 2019 (29)** | Retrospective cohort | Determine the effect of presence/absence of MMC sac on fetall talipes | Prenatal diagnosis | 18-24 weeks | NA | NA | NA | NA | US specialists |
| **Ramin, 2002 (25)** | Cross-sectional (retrospective analysis) | Assess the association between prenatal ultrasound markers and postnatal neurological outcomes in fetuses with MMC | Prenatal diagnosis | 19-22 weeks | Yes | Yes | Toshiba SSA-270, ATL5000 | 3.75-5.2-MHz transducer | NA |
| **Wax, 2009 (31)** | Cross-sectional (retrospective analysis) | Determine the incidence of pointed deformity of occipital horns of lateral ventricles in fetuses with and without MMC | Prenatal diagnosis | 19.2 weeks* | NA | NA | NA | NA | US specialists |
| **Wong, 2009 (33)** | Cross-sectional (retrospective analysis) | Determine the incidence of interhemispheric arachnoid cysts in fetuses with MMC | Prenatal diagnosis | 17-35 weeks | NA | NA | NA | 2- 4-MHz vector or 2- 6-MHz curved array transducer | Single US specialist |

Abbreviations: MMC, myelomeningocele; BPD, biparietal diameter; HC: head circumference; NA, data not available; US ultrasound

†Combison 330/530D MT, Voluson730 Expert/E8, Sonoace Accuvix, Hitachi EUB 6500, Siemens Elegra, Acuson Sequoia, GE Logiq 9, HDI 5000 Sono CT, Philips IU22, Aloka Prosound SSD, Aplio XG/500

*Average GA at diagnosis

**Table S2.** Quality appraisal for cohort study assessed by Newcastle-Ottawa scale

| Study name | Selection 1 | Selection 2 | Selection 3 | Selection 4 | Comparability | Outcome 1 | Outcome 2 | Outcome 3 | Summary |
|---|---|---|---|---|---|---|---|---|---|
| **Appasamy, 2006** (47) | 1 | 1 | 1 | 1 | 0 | 1 | 1 | 1 | Poor |
| **Fleurke-Rozema, 2014** (49) | 1 | 1 | 1 | 1 | 0 | 1 | 1 | 1 | Poor |
| **Maurice, 2020** | 1 | 1 | 1 | 0 | 1 | 1 | 1 | 1 | Good |
| **Munoz, 2019** (52) | 1 | 1 | 1 | 1 | 0 | 1 | 1 | 1 | Poor |
| **Oliver, 2019** (53) | 1 | 1 | 1 | 1 | 1 | 1 | 1 | 1 | Good |

**Table S3.** Quality appraisal for cross-sectional study assessed by modified Newcastle-Ottawa scale

| Study name | Selection 1 | Selection 2 | Selection 3 | Selection 4 | Comparability | Outcome 1 | Outcome 2 | Summary |
|---|---|---|---|---|---|---|---|---|
| **Bahlmann, 2015** (26) | 1 | 0 | 1 | 2 | 0 | 2 | 1 | Good |
| **Callen, 2008** (30) | 1 | 0 | 1 | 2 | 0 | 1 | 1 | Satisfactory |
| **Callen, 2009** (27) | 1 | 0 | 1 | 2 | 0 | 1 | 1 | Satisfactory |
| **Cuppen, 2015** (20) | 1 | 0 | 1 | 2 | 0 | 2 | 1 | Good |
| **D'Addario, 2008** (18) | 1 | 0 | 1 | 2 | 0 | 2 | 0 | Satisfactory |
| **Filly, 2010** (32) | 1 | 0 | 1 | 2 | 1 | 1 | 1 | Good |
| **Ghi, 2006** (51) | 1 | 0 | 1 | 2 | 0 | 2 | 0 | Satisfactory |
| **Ramin, 2002** (40) | 1 | 0 | 1 | 2 | 0 | 2 | 1 | Good |
| **Wax, 2009** (31) | 1 | 0 | 1 | 2 | 2 | 1 | 1 | Good |
| **Wong, 2009** (33) | 1 | 0 | 1 | 2 | 0 | 1 | 0 | Satisfactory |
