# Supplementary material for: Cranial findings detected by second‐trimester ultrasound in fetuses with myelomeningocele: a systematic review
Source: BJOG. 2021 Jan 3;128(2):366–74. doi: 10.1111/1471-0528.16496 (PMC8436766; doi:10.1111/1471-0528.16496)
Supplement: Supplementary file 2 — Appendix S1. Search strategy. Appendix S2. Characteristics of included studies and results. [file BJO-128-366-s011.pdf]

**Appendix S1.** Search strategy.

| **Search strategy (MESH terms and keywords)** |
|---|
| "spinal dysraphism" [MeSH] OR "arnold-chiari malformation" [MeSH] OR "meningomyelocele" [MeSH] OR spinal dysraphism OR spina bifida OR Arnold-chiari malformation OR meningomyelocele<br>AND<br>"ultrasonography, prenatal/" [MeSH] OR ultrasound OR sonography OR ultrasonography OR (2D or 3D or 4D) adj2 ultrasound OR (2D or 3D or 4D) adj2 sonography OR (2D or 3D or 4D) adj2 ultrasonography<br>AND<br>"pregnancy, high-risk" [MeSH] OR "pregnancy trimester, second/ or pregnancy trimester, third/" [MeSH] OR "fetus" [MeSH] OR "Prenatal care" [MeSH] or pregnan* adj4 trimester OR f?etal OR f?etus |

**Appendix S2.** Characteristics of included studies and results.

| **Characteristics of included studies** |
|---|
| **Participant characteristics**<br>- Maternal age<br>- Gestational age at the time of evaluation<br>- Associated anomalies or chromosomal abnormalities<br>- Diagnostic confirmation (availability of postnatal examination or post-termination of pregnancy autopsy) |
| **Technical aspects**<br>- Ultrasound machine<br>- Ultrasound probe<br>- Levels of experience and numbers of sonographers |
| **Study methodology**<br>- First author name<br>- Title of the paper<br>- Year of publication<br>- Study design (cohort/cross-sectional studies/randomised control trial)<br>- Patient enrolment (selective or non-selective) |
| **Study results**<br>- Definition of detected intra-cranial findings<br>- Total number of examination and crude number of each intra-cranial finding |
